# Surface display of a manganese-binding domain enhances production and stress resistance in *Bacillus subtilis* spores

**DOI:** 10.3389/fmicb.2025.1669207

**Published:** 2025-10-09

**Authors:** Nhi N. Y. Nguyen, Tuom T. T. Truong, Dong Van Nguyen, Trang T. P. Phan, Hoang Duc Nguyen

**Affiliations:** ^1^Center for Bioscience and Biotechnology, VNUHCM-University of Science, Ho Chi Minh City, Vietnam; ^2^Vietnam National University, Ho Chi Minh City, Vietnam; ^3^Faculty of Chemistry, VNUHCM-University of Science, Ho Chi Minh City, Vietnam

**Keywords:** *Bacillus subtilis*, spore, sporulation, MntC, manganese binding domain, surface display, vaccine delivery, stress resistance

## Abstract

**Introduction:**

*Bacillus subtilis* spores are widely used as platforms for antigen display due to their stability and safety. However, the potential impact of surface-expressed functional proteins, such as metal-binding antigen proteins, on spore physiology remains largely unexplored. This study investigated the effects of the surface-expressed manganese-binding domain of manganese transport protein C (MntC) from *Staphylococcus aureus* on spore development and stress resistance.

**Methods:**

A recombinant *B. subtilis* strain, BsHT2380, was engineered by double cross-over integration of P*cotB-cotB-mntC* at the *amyE* locus, confirmed by PCR. MntC expression on the spore surface was verified via western blot, spore ELISA and confocal fluorescence microscopy. BsHT2380 spores exhibited increased manganese accumulation compared to controls, as measured by flame atomic absorption spectroscopy (F-AAS). EDTA treatment confirmed that the bound Mn^2+^ was surface-associated. Correlation between Schaeffer-Fulton staining and CFU counts indicated that Mn^2+^ accumulation enhanced spore production efficiency.

**Results:**

The BsHT2380 strain produced 71% mature spores by 48 hours, with spore levels remaining stable from 48 to 72 hours, suggesting this period represents the peak of sporulation. Importantly, BsHT2380 spores displayed enhanced resilience, with significantly higher survival rates under lysozyme (73%) and wet heat (70%) stress compared to control strains.

**Discussion:**

These findings demonstrated that surface-expressed manganese binding domain could modulate spore physiology, improving both production and resistance, and highlight the potential of surface-displayed proteins in spore-based biotechnological applications, particularly recombinant spore-based vaccines that combine immunogenic antigen presentation with enhanced structural robustness.

## 1 Introduction

*Bacillus subtilis* is a well-established microbial cell factory for recombinant protein production, offering several advantages including GRAS (generally regarded as safe) status, well-characterized genome, and suitability for large-scale fermentation (Su et al., 2020). Under stressful environmental conditions, *B. subtilis* undergoes sporulation, forming metabolically dormant and highly resistant endospores. These spores possess a robust multilayered coat that protects against harsh environmental stresses, making them attractive platforms for surface display of heterologous proteins. Spore-based display system has been exploited for diverse applications such as vaccine delivery, enzyme immobilization, biosensing, and the development of probiotic and therapeutic formulations (Zhang et al., 2020).

Advances in *B. subtilis* spore surface display technologies have enabled the stable anchoring of functional proteins to spore coat layers through both recombinant and non-recombinant strategies (Guoyan et al., 2019). Recombinant approaches typically involve genetic fusion of target proteins to spore coat proteins such as CotB, CotC, or CotG, or to crust proteins such as CotZ or CotY, facilitating controlled expression during specific stages of sporulation (Guoyan et al., 2019). Non-recombinant methods, on the other hand, rely on passive adsorption or chemical cross-linking of purified proteins onto the spore surface (Guoyan et al., 2019). These techniques have expanded the versatility of *B. subtilis* spores as platforms, enabling them to interact directly with environmental factors or to stimulate immune responses. Despite these advances, most studies have predominantly focused on the catalytic or immunological activity of the displayed proteins, while the potential influence of these surface-displayed proteins on the physiology and developmental processes of the host spore itself has remained largely unexplored.

MntC is a highly conserved manganese-binding lipoprotein from *Staphylococcus aureus*. Structurally, MntC is characterized by a long α-helix connecting two domains, which is proposed to restrict metal dissociation and contribute to the high-affinity manganese binding, observed in the low nanomolar range (Gribenko et al., 2013). The immunogenic potential of MntC is well recognized, and it has been included as a candidate in several vaccine formulations (Anderson et al., 2012; Yu et al., 2018; Ahmadi et al., 2021). However, its physiological influence when expressed on the spore surface of *B. subtilis* is unknown.

Among essential micronutrients, manganese plays a particularly critical role in the sporulation of *Bacillus* species (Charney et al., 1951). It contributes to oxidative stress defense, enzymatic function, and spore coat formation. During the initial stage, Mn^2+^ accumulates in the forespore, functioning as a cofactor for SpoIIE serine phosphatase (Jakubovics and Jenkinson, 2001). SpoIIE plays a role in the normal formation of the asymmetric septum during sporulation and regulates the activity of the transcription factor sigma F, which is responsible for directing early forespore-specific gene expression (Barák et al., 1996). In addition, Mn-dependent superoxide dismutase (SOD) is involved in the proper assembly of the spore coat (Henriques et al., 1998), and manganese is also required for phosphoglycerate mutase activity during carbohydrate metabolism (Oh and Freese, 1976). In the absence of manganese, cells accumulate intracellular 3-phosphoglyceric acid (3PGA), a metabolic intermediate, and fail to initiate or complete the sporulation process (Oh and Freese, 1976).

Given the essential role of manganese in sporulation and stress resistance, this study explores whether surface expression of a manganese-binding protein, specifically MntC, can influence spore development and resistance beyond its established function as a vaccine antigen. To our knowledge, no previous studies have addressed whether surface-displayed metal-binding proteins can actively modulate the physiological or developmental characteristics of *B. subtilis* spores.

In this work, we constructed a recombinant *B. subtilis* strain expressing the manganese-binding domain of MntC on the spore surface through translational fusion with the coat protein CotB. The successful surface localization of MntC was confirmed, followed by the evaluation of spore production efficiency and resistance to both physical and chemical stress conditions, specifically wet heat and lysozyme exposure. These findings reveal an unrecognized functional role for surface-displayed protein in modulating spore biology and suggest new strategies for enhancing the performance of spore-based platforms in biotechnological applications.

## 2 Materials and methods

### 2.1 Construction of plasmid and strain

Competent *E. coli* OmniMax and *B. subtilis* cell*s* were prepared using the procedures described (Green et al., 2012; Smith, 1991). The *B. subtilis* control strain HT800F (patent no. VN103078; Vietnam National Office of Intellectual Property, 2024)) was from our lab stock collection, which originated from WB800N (Nguyen et al., 2011; Jeong et al., 2018).

The vector pHT2380 was designed as a shuttle vector for double-crossover integration into *B. subtilis*. It contains the fusion sequence of PcotB-cotB-mntC, which was synthesized by GenScript. To generate the recombinant *B. subtilis* strain, we introduced the vector pHT2380 to competent cells of HT800F strain. Colonies were selected on an agar plate with neomycin added at 25 μg/mL, then confirmed by colony PCR using three pairs of primers. Primer information was listed in Table 1. *B. subtilis* new strain BsHT2380 was stored at −80 °C.

**Table 1:** Primers used in this study.

Primers	Sequence (5′–3′)	Purpose	Amplicon length (bp)
ON469	GGCGTTCTGTTTCTGCTTCG	PCR for integration checking at *amy5E*	1,100
ON1479	GTCTGGTCAACTTTCCGACTCTG		
ON470	AACCCGCTCCGATTAAAGCTAC	PCR for integration checking at *amy3E*	1,172
ON859	GGAGCCATCCGCAATTTGAA		
ON2303	GCTCATCAAAATCATCTAAACGATCAC	PCR for integration checking at the region between *amy5E* and *amy3E*	1,022
ON1666	TACCTGCAGGGAATTCCGGGGACGTTATTTTTCAAATTGC		

### 2.2 Preparation of *B. subtilis* spores

*Bacillus subtilis* colonies were cultured directly in 400 mL of Difco Sporulation Medium (DSM) at 33 °C to induce the sporulation. The biomass was collected at different time points by centrifugation at 13,000 × *g* for 1 min. The biomass was treated with lysozyme (10 mg/mL) at 37 °C for 1 h to eliminate vegetative cells, followed by centrifugation at 13,000 × *g* for 1 min and four washes with 1 × PBS to obtain purified endospores. Samples were stored in PBS 1X with 10% glycerol at −80 °C for the following experiments.

To determine the percentage of sporulation, the colony-forming unit (CFU) number of samples taken from the culture before and after lysozyme treatment was counted using serial dilution and plate spread techniques. The experiment was performed in triplicate. The sporulation rate was calculated as follows:

$\begin{array}{l} \text{Percentage of sporulation}=\text{Number of endospores}/\\ \text{Total number of}\;\\ \text{endospores and}\;\\ \text{vegetative cells} \end{array}$

### 2.3 Schaeffer and Fulton’s method

The Schaeffer–Fulton method is a differential staining technique developed to visualize highly resistant bacterial endospores (Schaeffer and Fulton, 1933). A 20 μL volume of either biomass (cells and spores) or purified spores after 48 h of cultivation was smeared onto glass slides. The samples were heat-fixed and stained with 5% malachite green for 5 min, followed by cooling and rinsing with water to remove excess dye. Subsequently, 2.5% safranin O was applied for 30 s, after which the slides were rinsed with water and air-dried. The results were then examined under a light microscope.

### 2.4 Fluorescence confocal microscopy

The purified endospores were fixed with 4% formaldehyde for 20 min, washed three times with PBS 1X, and then incubated with blocking buffer (PBS-T with 5% skim milk) for 30 min. The primary antibody for MntC developed in Swiss mice by our research group was used at a ratio of 1:1000 for 1 h, gently shaking. After the washing steps, the sample was incubated with the secondary antibody Goat anti-Mouse IgG (H+L) Cross-Adsorbed Secondary Antibody, Alexa Fluor™ 488 (Invitrogen, A-11001) at the concentration of 1:1000 and washed eventually. Finally, a 5 μL sample was spread on the slide, added 5 μL mounting media, and then covered with a coverslip. The result was read by Confocal Microscopy (Nikon AX) and analyzed by NIS-Element Viewer Software.

### 2.5 Spore ELISA

The spore ELISA was performed as described in our previous publication (Nguyen et al., 2021). Briefly, the purified spores were resuspended in coating buffer (100 mM NaHCO_3_; pH 9.6) to the OD_600nm_ of 4, then coated onto a 96-well plate (Thermo Scientific™ Nunc™ MicroWell™ 96-Well Microplates) at 50 μL per well. After incubation, washing, and blocking, the primary MntC-antibody, as the same used for Fluorescent Confocal Microscopy, was used at a dilution ratio of 1:10,000. The second antibody was anti-mouse IgG—peroxidase antibody produced in rabbits (whole molecule) (Sigma, A9044–2 mL), used at a ratio of 1:40,000. The signal was developed by TMB Liquid Substrate for ELISA (Sigma). Absorbance values were measured at a wavelength of 450 nm in a CLARIOstar plate reader. Each sample was tested in triplicate, with results expressed as mean ± SD. Statistical significance was determined using a *t*-test performed with GraphPad Prism (USA).

### 2.6 Western blot

Purified spores were adjusted to an optical density of 5 at 600 nm, pelleted by centrifugation at 13,000 × *g* for 1 min, and resuspended in spore protein extraction buffer (50 mM Tris–HCl, pH 6.8, 1% SDS, and 50 mM DTT). The suspension was incubated at 65 °C for 1 h to extract proteins, and the supernatant containing the extracted proteins was collected after centrifugation. Proteins were separated by SDS-PAGE and transferred onto a nitrocellulose membrane. The membrane was blocked in PBS–0.1% Tween™ (PBS-T) containing 5% (w/v) skim milk (Oxoid™), and incubated overnight at 4 °C with a primary anti-MntC antibody, generated in Swiss mice by our group, diluted 1:20,000 in blocking buffer. After washing, the membrane was incubated for 1 h at room temperature with a rabbit anti-mouse IgG peroxidase-conjugated secondary antibody (whole molecule, Sigma A9044), diluted 1:160,000 in blocking buffer. Following final washes, protein expression was visualized using TMB substrate, and the colorimetric signal was recorded. Protein sizes were estimated using the PageRuler™ Plus Prestained Protein Ladder (Thermo, 26619).

### 2.7 Flame atomic absorption spectrometry (F-AAS)

The biomass of *B. subtilis* spores was washed with deionized water, collected at an OD_600nm_ of 5, and then mixed with 0.5 mL of nitric acid 65% (Merck) and heat-treated at 80 °C in a water bath for 10 min. When dissolved entirely, the sample was added precisely with 0.5 mL of deionized water and diluted appropriately. A flame atomic absorption spectrometer (Shimadzu AA6300, Japan) was used to determine the Mn^2+^ concentration.

### 2.8 Lysozyme and wet heat resistance test

Purified endospores were incubated with lysozyme at the concentration of 15 mg/mL at 37 °C for 1 h, shaking at 200 rpm. Spores were incubated with distilled H_2_O at a ratio of 1:1 at 85 °C for 30 min to test the wet heat resistance. All samples were then centrifuged at 13,000 ×*g* for 1 min and washed with PBS 1X. The serial dilution method was applied to determine the number of survival spores.

## 3 Results

### 3.1 Construction of *B. subtilis* HT2380 for displaying the manganese-binding domain

**Figure 1:**
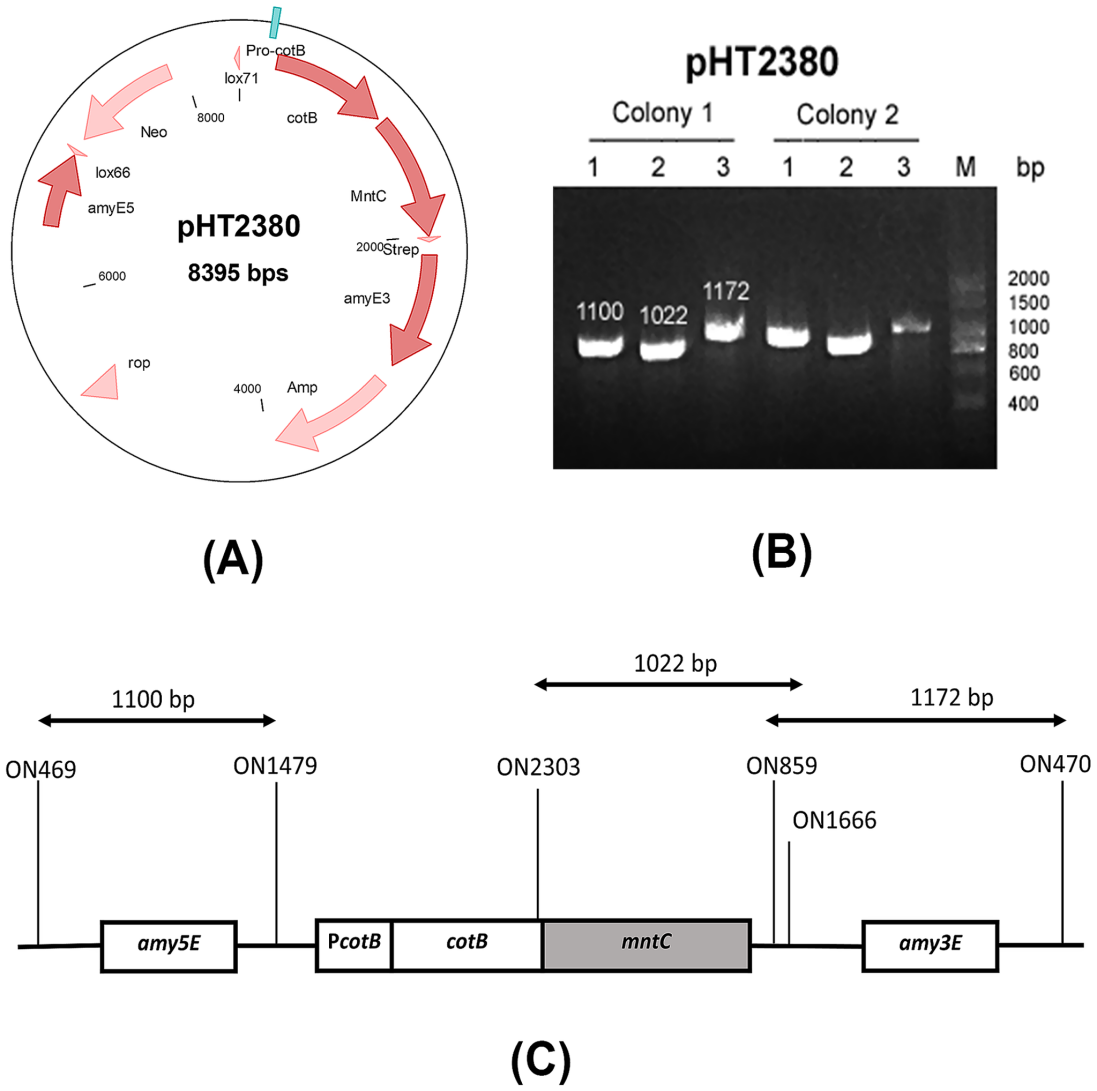
Construction of the BsHT2380 strain **(A)** schematic representation of the plasmid pHT2380, **(B)** agarose gel electrophoresis of colony PCR products using three primer sets, verifying the successful construction of BsHT2380, **(C)** diagram showing the primer binding sites used in colony PCR to confirm double-crossover integration at the *amyE3* and *amyE5* loci.

The recombinant plasmid pHT2380 was constructed using pHT2304 (Nguyen et al., 2021), a shuttle vector backbone derived from pCotB-CL (Iwanicki et al., 2014). This plasmid contains key elements for cloning and chromosomal integration, including the *E. coli* ColE1 origin of replication, multiple cloning sites, ampicillin and neomycin resistance genes for selection, and homologous regions (*amyE3* and *amyE5*) for double-crossover integration at the *amyE* locus. In addition, pHT2380 features two mutant loxP sites (*lox66* and *lox71*) for Cre-lox-based elimination of antibiotic resistance markers, and a *cotB-mntC* fusion for spore surface display under the control of the native P*cotB* promoter. A schematic map of the pHT2380 construct was shown in Figure 1A.

The vector pHT2380 containing the manganese-binding domain of MntC was introduced into *B. subtilis* HT800F competent cells via natural transformation. Homologous recombination at the *amyE* locus facilitated stable chromosomal integration of the *cotB-mntC* cassette. This genomic integration ensures the inserted gene remains stably maintained in the chromosome without requiring continuous antibiotic selection pressure (Härtl et al., 2001). Transformants were selected on antibiotic-containing agar and screened by colony PCR using three primer pairs: two targeting the 5′ and 3′ flanking regions of the *amyE* locus, and one specific for the *cotB-mntC* fusion. The primer binding sites were illustrated in Figure 1C. PCR analysis (Figure 1B) confirmed the successful integration, yielding the expected amplicon sizes of 1,100 bp, 1,022 bp, and 1,172 bp for the respective primer sets. A confirmed colony was subsequently cultured in LB medium and stored in 15% glycerol at −80 °C. The recombinant strain was designated BsHT2380.

### 3.2 Surface display of MntC on BsHT2380 spore

The BsHT2380 strain was engineered to express a CotB-MntC fusion protein during sporulation, driven by the native *PcotB* promoter, to facilitate surface localization of the manganese-binding domain on the spore coat. BsHT2380 and two control strains, BsHT800F (parental strain) and BsHT2304 (Nguyen et al., 2021) (harboring a similar construct without the target gene *mntC*), were cultured in Difco Sporulation Medium (DSM) for 48 h to induce spore formation. To confirm the successful display of MntC, spores were collected for western blot, spore ELISA and immunofluorescence microscopy to verify the surface display of MntC.

**Figure 2:**
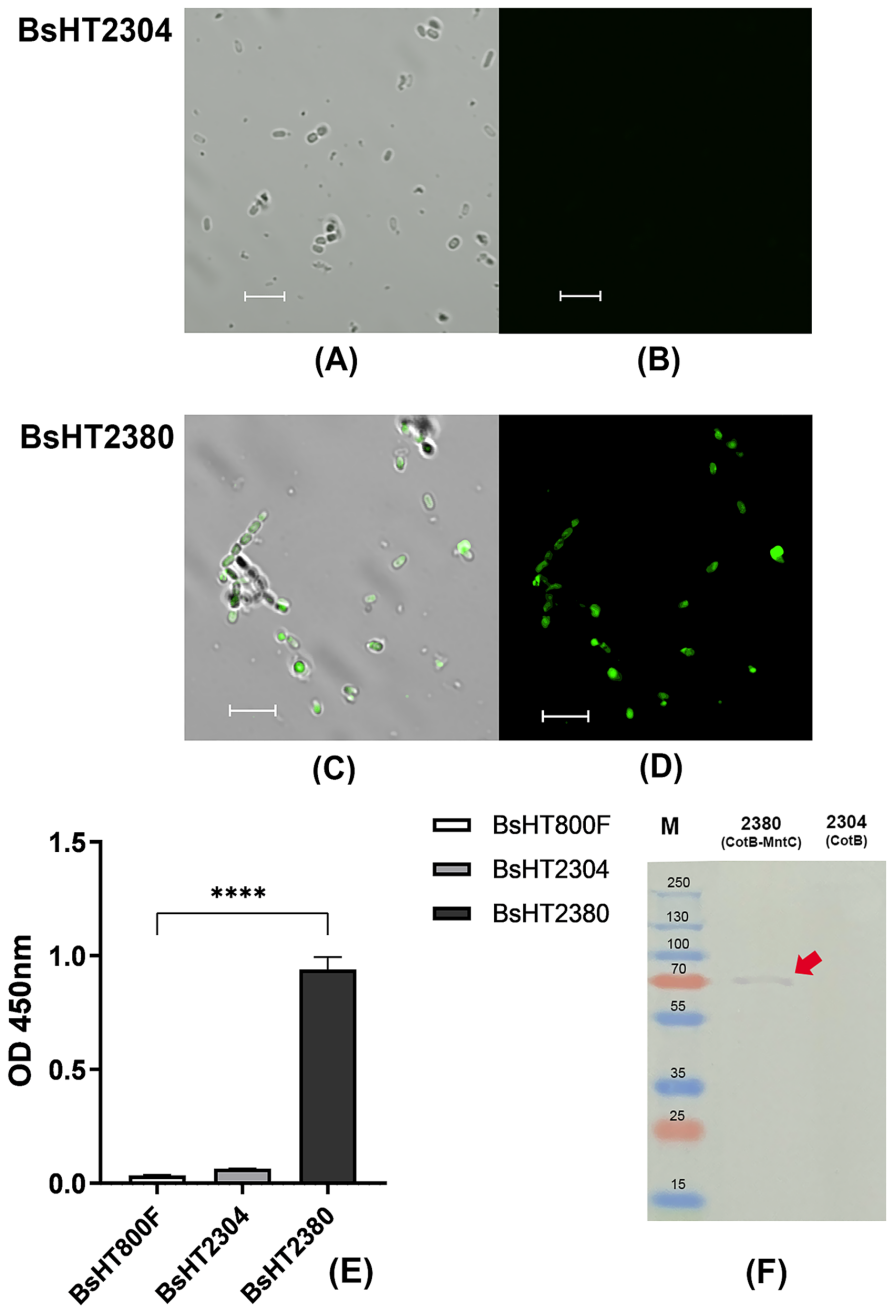
Confirmation of surface display of MntC on BsHT2380 spores. **(A–D)** Confocal microscopy images of immunostained spores. Spores were labeled with anti-MntC primary antibody followed by Alexa Fluor 488-conjugated secondary antibody. **(A,C)** Overlay images of transmitted light and fluorescence signals for BsHT2304 (CotB) and BsHT2380 (CotB-MntC), respectively. **(B,D)** Fluorescence signals only for BsHT2304 and BsHT2380, respectively. **(E)** Quantification of MntC display on spore surfaces by spore ELISA. White: BsHT800F (parental control), gray: BsHT2304 (CotB-only control), black: BsHT2380. (****: *p* ≤ 0.0001). Scale bar 5 μm. **(F)** Western blot analysis of BsHT2377 and the control strain BsHT2304.

The Western blot results shown in Figure 2F revealed a distinct band in the BsHT2380 sample, whereas no band was observed in the control BsHT2304, demonstrating the specific recognition of MntC expressed in BsHT2380 by the anti-MntC antibody. The expected molecular mass of MntC alone is 32.8 kDa, while the CotB-MntC fusion protein is predicted to be 66 kDa. A visible band appeared at approximately 70 kDa, confirming the stable expression of the CotB-MntC fusion protein.

In spore ELISA, the whole endospores, suspended in a coating buffer at an OD_600nm_ value of 4, were coated onto a 96-well plate as an antigen factor. The next steps were basically the indirect ELISA, in which the released signal proved the positive interaction between the spores and the specific anti-MntC antibody. Results in Figure 2E demonstrated a statistically significant increase in MntC expression on the BsHT2380 sample (*p* ≤ 0.0001). This strong signal suggested a successful interaction between the surface proteins of BsHT2380 and the anti-MntC antibody, confirming the expression of the target protein, MntC, by the spores immobilized on the plate.

To further confirm the presence of MntC on the spore surface, we performed immunofluorescence labeling. Spores were incubated with an anti-MntC primary antibody and a fluorescent secondary antibody, followed by visualization using confocal fluorescence microscopy. Figure 2D showed that the BsHT2380 sample emitted clear and high fluorescence signals. In the picture under the transmitted light channel (TD) (Figure 2C), they appeared as intact objects with thick walls and approximately 1.5 μm in length, which is the morphological characteristics of *B. subtilis* endospores. Besides, BsHT2304, the control sample that expressed CotB only, was observed with similar spore morphology images through the TD channel (Figure 2A) but lacked any fluorescence signal (Figure 2B).

Taken together, the distinct fluorescence observed by confocal microscopy, along with the Western blot and spore ELISA results, conclusively demonstrated MntC expression and its anchoring by CotB on the outer spore coat of BsHT2380.

### 3.3 Manganese accumulation in the spore

We next evaluated whether the manganese-binding activity of recombinant MntC displayed on *B. subtilis* spores was functionally effective by measuring manganese ion concentrations in BsHT2380 compared to the parental strain BsHT800F. Both strains were sporulated under the same conditions and harvested at two time points: 48 and 72 h. Spores were treated with lysozyme to remove residual vegetative cells, with or without the addition of EDTA, and then subjected to ion quantification. Manganese levels were normalized to calcium content (Mn/Ca ratio), as Ca-DPA (calcium-dipicolinic acid) in the spore core remains stable and unaffected by surface treatments, including EDTA, under standardized sporulation conditions. In contrast, manganese associated with the spore coat can be removed by EDTA, making calcium a reliable internal reference for spore quantity. To compare strains, the relative Mn/Ca ratio (reported as “Mn/Ca vs. Control” in Table 2) was calculated by dividing the Mn/Ca value of BsHT2380 by that of the control strain. The percentage reduction by EDTA (reported as “% Mn reduction by EDTA” in Table 2) was defined as the decrease in the Mn/Ca ratio of EDTA-treated samples relative to untreated samples, expressed as a percentage of the untreated value.

**Table 2:** Manganese accumulation in *B. subtilis* spores HT2380 and HT800F with and without EDTA treatment.

Time (h)	Strain	Treatment	Mn/Ca ratio	Mn/Ca vs. control	Mn/Ca Fold Change (H₂O vs EDTA)	% Mn reduction by EDTA
48	HT2380	H₂O	0.152	1.23	1.84	45.64%
	HT800F	H₂O	0.124	1.00	1.35	25.66%
	HT2380	EDTA	0.082	0.90	–	–
	HT800F	EDTA	0.092	1.00	–	–
72	HT2380	H₂O	0.176	0.99	1.87	46.60%
	HT800F	H₂O	0.174	1.00	1.28	21.84%
	HT2380	EDTA	0.094	0.69	–	–
	HT800F	EDTA	0.136	1.00	–	–

As shown in Table 2, both BsHT2380 and control spores exhibited a reduction in Mn/Ca ratio upon EDTA treatment; however, the decrease was greater in BsHT2380, 45.64 and 46.6% at 48 and 72 h, respectively, compared to 25.66 and 21.84% in the control. This reduction indicated that MntC actively bound manganese on the spore surface, and that EDTA was capable of displacing it. These findings suggested that while wild-type spores retain a modest amount of surface-bound manganese, likely through nonspecific interactions, the presence of surface-displayed MntC enhanced manganese accumulation on the spore coat, making it more susceptible to EDTA-mediated removal.

The manganese content in spore samples treated with lysozyme and water, without EDTA, also revealed differences between strains. At 48 h, the Mn/Ca ratio in BsHT2380 was 1.23-fold higher than in BsHT800F, indicating an enhanced ability of BsHT2380 to bind and accumulate Mn^2+^ at this time point. However, by 72 h, this difference diminished, and the Mn/Ca ratios between the two strains became comparable. This suggests that the manganese-binding capacity of BsHT2380 is transient, being most evident during the early phase of cultivation after sporulation onset and diminishing as cultivation progresses, possibly due to saturation of surface MntC binding sites or reduced accessibility of MntC during spore maturation.

To further investigate whether the observed increase in Mn^2+^ accumulation was associated with improved spore production efficiency, we next examined spore formation kinetics and spore resistance in BsHT2380 compared to control strains.

### 3.4 Impact on sporulation kinetics

To evaluate the effect of MntC on sporulation progression, the biomass of *B. subtilis* strains BsHT800F, BsHT2304, and BsHT2380 was harvested at 48 h post-inoculation and subjected to Schaeffer–Fulton staining. This method distinguishes different cellular forms based on different staining properties: vegetative cells appear pink-red due to Safranin O uptake, mature endospores stain blue–green with Malachite Green, and prespores are observed as pink cells containing internal green-stained endospores.

**Figure 3:**
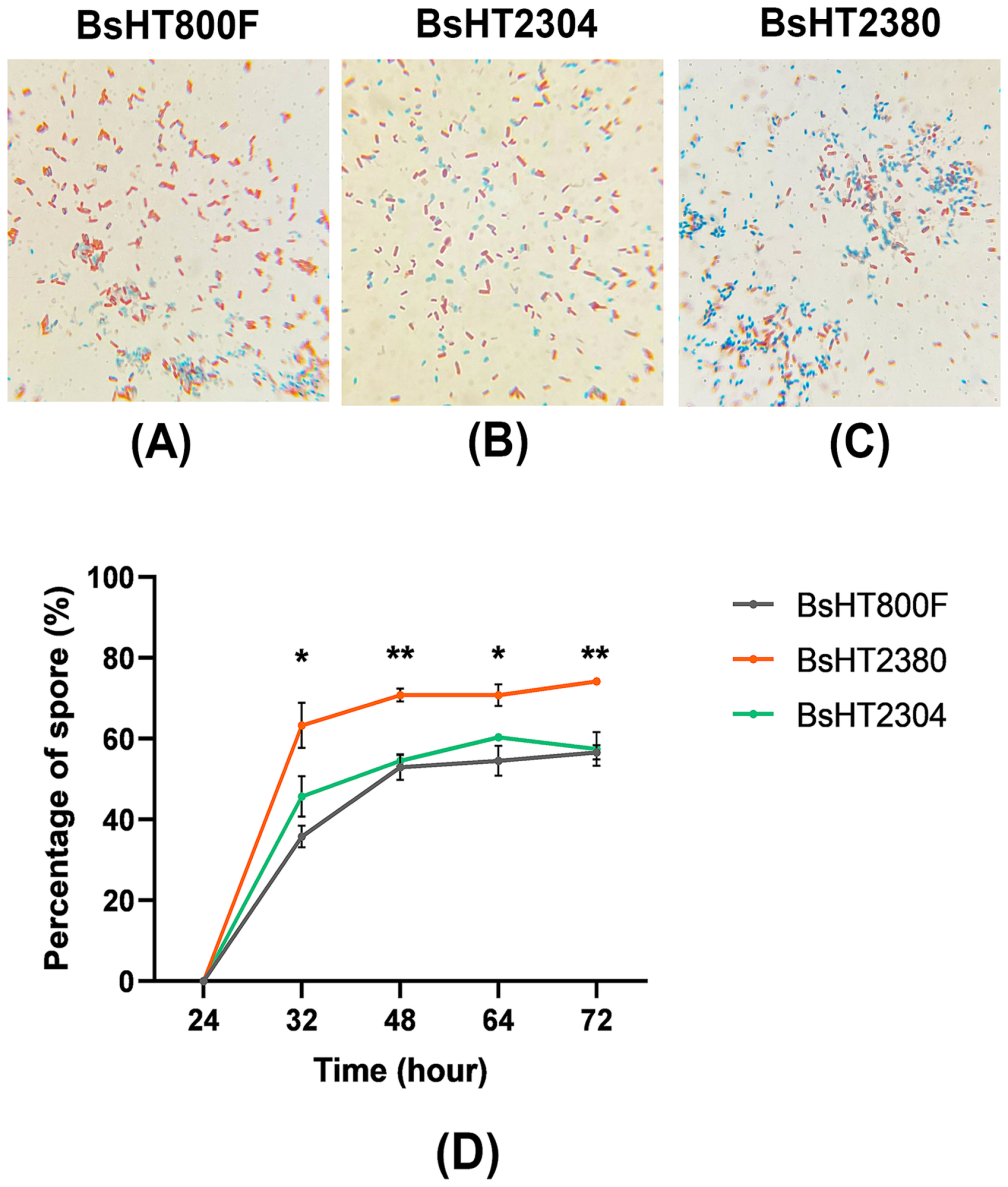
Sporulation efficiency of *B. subtilis* strains following 48 h of sporulation. **(A–C)** Schaeffer–Fulton staining of culture samples after 48 h for BsHT800F (−), BsHT2304 (CotB), and BsHT2380 (CotB-MntC). Vegetative cells: red–pink, spores: blue–green. **(D)** Percentage of mature spores over time. Gray: BsHT800F (parental control); green: BsHT2304 (CotB-only control); orange: BsHT2380. (*: *p* ≤ 0.05, **: *p* ≤ 0.01).

Microscopy images shown in Figures 3A–C illustrate the presence of vegetative cells, prespores, and mature spores in all three strains. In these representative images, the recombinant strain BsHT2380 appeared to contain more fully stained mature endospores compared to the control strains BsHT800F and BsHT2304. This qualitative observation suggests that BsHT2380 may generate a higher number of spores.

To investigate the impact of MntC expression on sporulation kinetics, the percentage of mature spores was quantified at five distinct time points. Spores were isolated, serially diluted, and counted via colony-forming unit (CFU) analysis. As shown in Figure 3D, sporulation initiation was observed around 24 h post-inoculation across all strains. By 32 h, BsHT2380 exhibited a significantly higher proportion of mature spores (~63%) compared to the control strains BsHT800F and BsHT2304, both of which remained below 50% (*p* < 0.05). At 48 h, BsHT2380 reached 71% spore formation, significantly exceeding BsHT800F (53%) and BsHT2304 (55%) (*p* < 0.01). From 48 to 72 h, spore percentages remained relatively stable in all strains; however, BsHT2380 consistently maintained the highest spore proportion (74%), compared to BsHT800F and BsHT2304 (both 57%). The minimal difference between the two control strains indicated that the plasmid backbone in BsHT2304, lacking the *mntC* insert, did not impact the sporulation.

Collectively, these findings demonstrated that surface expression of MntC in BsHT2380 enhanced the kinetics and efficiency of spore production in *B. subtilis*, resulting in a consistently higher proportion of mature spores. The relative plateau in spore percentages beyond 48 h across all strains suggested that this time point may represent the peak of sporulation under the tested conditions.

### 3.5 Impact on resistance characteristics to lysozyme and wet heat

**Figure 4:**
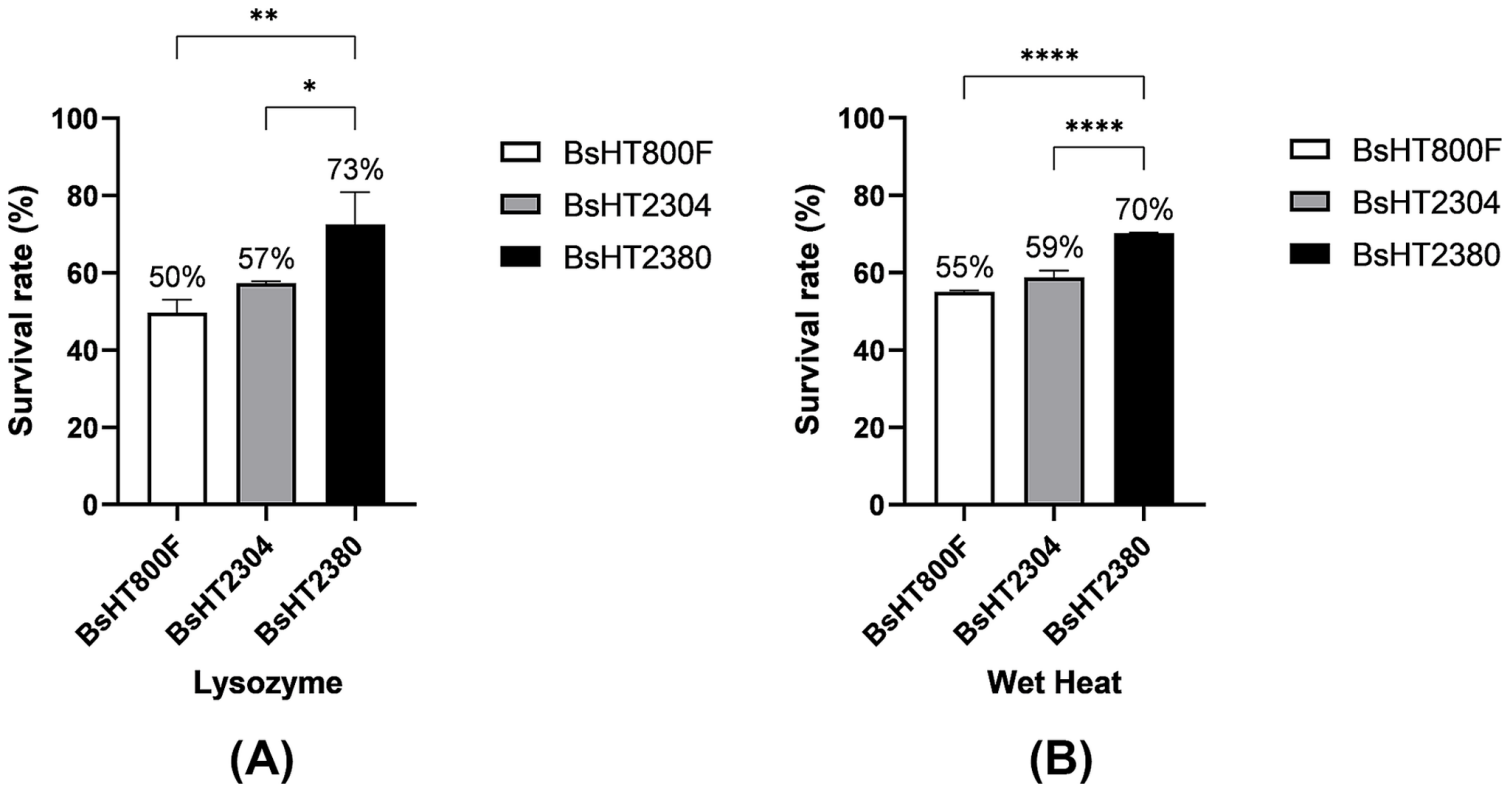
Resistance of *B. subtilis* spores to stress conditions. Survival percentage of spores after treatment with **(A)** lysozyme and **(B)** wet heat. White: BsHT800F (parental control); gray: BsHT2304 (CotB-only control); black: BsHT2380. (*: *p* ≤ 0.05, **: *p* ≤ 0.01, ***: *p* ≤ 0.001,****: *p* ≤ 0.0001).

To evaluate whether the BsHT2380 strain retained or enhanced its resistance characteristics, purified spores were subjected to lysozyme and wet heat treatments. Spore survival was quantified by CFU counting and expressed as a percentage relative to the total input spores under each treatment condition (Figure 4). The results showed that BsHT2380 exhibited significantly improved resistance compared to both the parental strain BsHT800F and the plasmid control strain BsHT2304 under the same treatment conditions. Upon exposure to lysozyme, BsHT2380 spores exhibited significantly higher survival (73%) compared to BsHT800F (50%, *p* ≤ 0.01) and BsHT2304 (57%, *p* ≤ 0.05). In response to wet heat, BsHT2380 also demonstrated enhanced resistance, with a survival rate of 70%, which was significantly higher than both BsHT800F (55%, *p* ≤ 0.01) and BsHT2304 (59%, *p* ≤ 0.0001). These results suggested that surface display of MntC enhanced spore resistance to both chemical (lysozyme) and physical (wet heat) stressors.

## 4 Discussion

In this study, we engineered *B. subtilis* to display the manganese-binding domain of MntC from *S. aureus* on the surface of its endospores. Our strategy involved: (i) constructing a recombinant strain based on *B. subtilis* HT800F (patent no. VN103078; Vietnam National Office of Intellectual Property, 2024), which enables stable expression of recombinant proteins and eliminates the repeated DNA sequences present in *B. subtilis* WB800 and WB800N strains; (ii) integrating the *mntC* gene into the chromosome via double-crossover recombination to prevent gene loss and minimize antibiotic dependence; and (iii) generating a translational fusion of MntC with the spore coat protein CotB to ensure accurate expression and stable surface display of MntC. The successful surface display of the fusion protein on *B. subtilis* spores was evidenced by the visible band in the Western blot, the strong signal observed in spore ELISA and the clear, intense fluorescence detected by confocal microscopy. This surface expression expands the potential of *B. subtilis* spores as a platform for presenting *S. aureus* antigens. Previous studies have reported the successful display of other *S. aureus* antigens, such as mutant α-toxin (Nguyen et al., 2021) and segments of Panton-Valentine leucocidin (Nguyen et al., 2025), on the surface of *B. subtilis* spores. Here, we first demonstrated the effective display of *S. aureus* MntC on the spore surface.

To evaluate the manganese-binding activity of BsHT2380, we quantified Mn^2+^ levels in purified spore samples and normalized the values to calcium content to ensure consistent comparison across strains. Calcium ions, primarily chelated with dipicolinic acid (DPA) in the spore core, are unaffected by surface treatments and remain stable under standardized sporulation conditions (Setlow et al., 2006). Since all strains (BsHT800F, BsHT2304, and BsHT2380) were derived from the same genetic background and cultured under identical conditions, variability in Ca-DPA (calcium-dipicolinic acid) levels was minimized. This approach is supported by prior studies employing calcium-normalized elemental ratios using time-of-flight secondary ion mass spectrometry (TOF-SIMS), where ^40^Ca served as a reliable internal standard for elemental comparison in *B. subtilis* spores (Cliff et al., 2005). Manganese content was assessed in both untreated and EDTA-treated spore samples to determine whether MntC enhances surface Mn^2+^ retention. BsHT2380 spores exhibited a markedly greater reduction in Mn/Ca ratio following EDTA treatment compared to control strains, supporting that MntC facilitated manganese binding on the spore surface, and that this bound Mn^2+^ was susceptible to chelation. A moderate decrease in Mn/Ca ratio was also observed in the control strains, which may reflect the presence of weakly bound manganese on the native spore coat via nonspecific adsorption or low-affinity interactions with endogenous coat proteins (Granger et al., 2011). These findings suggested that while baseline levels of surface Mn^2+^ can be removed by EDTA in all strains, the enhanced accumulation observed in BsHT2380 was specifically attributed to the surface-displayed MntC. Structural studies of homologous MntC proteins from *S. aureus* have identified a single Mn^2+^ binding site within its metal coordination pocket (Gribenko et al., 2013). MntC exhibits a high affinity for Mn^2+^ (Gribenko et al., 2013), making it highly efficient in capturing Mn^2+^ from the environment. Upon Mn^2+^ binding, the release of Mn^2+^ can occur through various mechanisms depending on physiological conditions and interactions with other proteins in the MntABC transporter system. One primary mechanism involves MntC interacting with MntB, triggering a conformational change that reduces Mn^2+^ affinity, thereby facilitating its transfer into the MntB-MntA transport system (Handke et al., 2018). However, when MntC from *S. aureus* is expressed in *B. subtilis*, the native MntABC transporter system is absent. Consequently, manganese captured by the manganese-binding domain of MntC remains localized on the spore surface, where it may exert physiological effects. Although MntC in BsHT2380 was expressed with CotB at the late stage of sporulation, and thus is unlikely to influence earlier events such as Spo0A activation or Mn-SOD activity during engulfment, its presence during coat formation could enhance Mn^2+^ availability for coat-associated enzymes. The Mn-dependent superoxide dismutase (SodA) has been implicated in spore coat assembly in *B. subtilis*. Disruption of the *sodA* gene has been shown to result in spores with altered coat layers (Henriques et al., 1998), suggesting that Mn^2+^ contributes to proper coat protein incorporation. In this context, the increased local concentration of Mn^2+^ at the spore surface, facilitated by the manganese-binding protein, may enhance sporulation efficiency either (i) directly, by improving coat assembly in MntC-expressing spores and thereby increasing the yield of fully developed spores, or (ii) indirectly, through a communal effect in which Mn^2+^ serves as a shared reservoir for neighboring sporulating cells. Furthermore, since Mn^2+^ binding to MntC is high-affinity but reversible (Gribenko et al., 2013), the gradual release of Mn^2+^ into the surrounding medium could increase extracellular manganese availability, supporting sporulation in the broader population. Nevertheless, the specific mechanisms by which surface-bound Mn^2+^ influences sporulation dynamics require further investigation. Collectively, the increased spore production rate observed in the BsHT2380 strain suggests that the expression of surface-displayed proteins like MntC can influence the spores’ developmental physiology, offering new insights into the feedback potential of engineered spore surfaces in *B. subtilis*.

*B. subtilis* spores naturally exhibit high resistance to environmental stressors due to their complex structure, comprising a multilayered coat protein, a dehydrated core, and protective molecules such as Ca-DPA complexes (McKenney et al., 2013). While lysozyme effectively degrades vegetative cell walls, its activity against dormant spores is limited by the spore coat, particularly the disulfide bond–rich proteins that restrict enzymatic access to the lysozyme-sensitive cortex (Gould and Hitchins, 1963). Similarly, the resistance to wet heat is primarily attributed to the dehydrated spore core, the presence of Ca-DPA, the integrity of the spore coat, and the mineral ion composition of the core (Setlow, 2006). Among these, core-localized Mn^2+^ alone appears to have limited effect on thermal resistance (Setlow, 2006; Sinnelä et al., 2019). In this study, spores of the recombinant strain BsHT2380 exhibited significantly greater survival following lysozyme and wet heat treatment compared to control strains. This enhanced resistance was likely due to increased surface-associated manganese, facilitated by the high-affinity Mn^2+^ binding capacity of the MntC protein. Manganese may contribute to the coat stability through its role as a cofactor for Mn-dependent enzymes such as SodA, which is implicated in maintaining the spore coat integrity. Moreover, previous studies have shown that the impact of Mn^2+^ on spore resistance varies across *Bacillus* species, suggesting a species-specific influence of manganese on spore robustness (Bender and Marquis, 1985; Granger et al., 2011; Sinnelä et al., 2019). Additionally, the expression of the CotB-MntC fusion protein may influence spore coat assembly and stability. CotB is a key structural protein of the outer spore coat, commonly utilized in spore surface display systems due to its ability to anchor heterologous proteins without compromising coat integrity. Similar spore display strategy using CotB fusion has demonstrated improved enzymatic stability and stress resistance, as shown by the immobilization of manganese peroxidase on *B. subtilis* spores for agricultural application (He et al., 2025). Although the expression of CotB in fusion with recombinant protein could theoretically disrupt normal coat architecture, the enhanced resistance observed in the BsHT2380 strain in this study suggests that the coat structure remained intact or was even enhanced. This indicates that the CotB-MntC fusion may contribute positively to spore structural integrity and resilience under stress conditions.

The improvements observed in the recombinant strain BsHT2380, particularly its stable and high-level surface display of MntC, enhanced spore production rate, and increased resistance to stress, have demonstrated the feasibility of utilizing *B. subtilis* spores as a multifunctional vaccine delivery platform. MntC has been validated in preclinical studies as an immunogenic and protective antigen against *S. aureus* infections, either as a single antigen (Anderson et al., 2012; Yu et al., 2018), in multivalent formulations (Begier et al., 2017), or as a fusion construct (Ahmadi et al., 2019). In this study, the successful expression and surface localization of MntC in BsHT2380 not only preserved the antigen’s immunoreactivity but also improved sporulation efficiency and spore resistance to physical and chemical stress factors. These findings suggest that MntC surface display may contribute to the overall physiological robustness of the recombinant spores. This dual advantage of antigen presentation and improved biological performance supports the potential of BsHT2380 as a platform for next-generation mucosal vaccines. BsHT2380 offers a versatile foundation for future antigen fusion constructs, enabling the co-display of MntC with additional protective antigens, whether derived from *S. aureus* or unrelated pathogens. Such multivalent vaccine formulations could leverage the high sporulation yield, structural resilience, and immunogenic potential of spores, while remaining cost-effective and scalable for large-scale production and global distribution. Further research is necessary to evaluate the immunogenicity and protective efficacy of MntC-displaying spores *in vivo*, including potential effects with other antigens or adjuvants in spore-based vaccine formulations. While immunogenicity studies are ongoing, the stable expression of MntC suggests that BsHT2380 may serve as a promising mucosal vaccine candidate against *S. aureus*. Beyond vaccines, *B. subtilis* spores have been widely explored as versatile platforms for the surface display or delivery of functional proteins, including enzymes, therapeutic peptides, and diagnostic markers, underscoring their broad biotechnological potential. The enhanced sporulation efficiency and stress resistance of BsHT2380 not only strengthen its utility in applications such as mucosal vaccination, enzyme delivery, and diagnostic tool development, but also offer practical advantages by reducing cultivation time, energy consumption, and processing costs.

In summary, we successfully engineered the *B. subtilis* strain BsHT2380 to display the manganese-binding domain of MntC on the spore surface, where it acted as a functional Mn^2+^ binder. This modification enhanced manganese binding, increased sporulation efficiency, and improved resistance to environmental stressors. These findings suggest that surface-displayed antigens on *B. subtilis* spores can serve dual roles: facilitating immune presentation and modulating spore physiology. With both functional and structural advantages, BsHT2380 represents a promising platform for the development of recombinant spore-based vaccines. This work underscores the broader potential of spore surface engineering to enhance spore utility through the targeted display of metal-binding proteins.

## Data Availability

The datasets presented in this study can be found in online repositories. The names of the repository/repositories and accession number(s) can be found in the article/supplementary material.
